# Neurological Dysfunction in Long COVID Should Not Be Labelled as Functional Neurological Disorder

**DOI:** 10.3390/v15030783

**Published:** 2023-03-18

**Authors:** Christina M. Van der Feltz-Cornelis, Andrew S. Moriarty, William David Strain

**Affiliations:** 1 Department of Health Sciences, Hull York Medical School, (HYMS), University of York, York YO10 5DD, UK; 2 Diabetes and Vascular Medicine Research Centre, Institute of Biomedical and Clinical Science, College of Medicine and Health, University of Exeter, Exeter EX2 5AX, UK

**Keywords:** Long COVID, functional, functional neurological disorder (FND), comorbidity, classification, motor symptoms, balance symptoms, neurological dysfunction

## Abstract

There have been suggestions that Long COVID might be purely functional (meaning psychological) in origin. Labelling patients with neurological dysfunction in Long COVID as having functional neurological disorder (FND) in the absence of proper testing may be symptomatic of that line of thought. This practice is problematic for Long COVID patients, as motor and balance symptoms have been reported to occur in Long COVID frequently. FND is characterized by the presentation of symptoms that seem neurological but lack compatibility of the symptom with a neurological substrate. Although diagnostic classification according to the ICD-11 and DSM-5-TR is dependent predominantly on the exclusion of any other medical condition that could account for the symptoms, current neurological practice of FND classification allows for such comorbidity. As a consequence, Long COVID patients with motor and balance symptoms mislabeled as FND have no longer access to Long COVID care, whereas treatment for FND is seldom provided and is ineffective. Research into underlying mechanisms and diagnostic methods should explore how to determine whether motor and balance symptoms currently diagnosed as FND should be considered one part of Long COVID symptoms, in other words, one component of symptomatology, and in which cases they correctly represent FND. Research into rehabilitation models, treatment and integrated care are needed, which should take into account biological underpinnings as well as possible psychological mechanisms and the patient perspective.

## 1. Introduction

Long COVID, also known as post-COVID-19 syndrome or post-acute sequelae of SARS-CoV-2 infection (PASC), is becoming widely recognized as a distinct disease entity [1]. The Office for National Statistics (ONS) in the UK estimates approximately 2.1 million individuals self-report the disease, with over half of them having had the condition for over one year [2], making Long COVID a major and persistent disease burden on healthcare systems worldwide [3,4]. There is an ongoing controversy about the prevalence rates of Long COVID. Reported prevalence rates vary greatly between countries, depending on whether or not they allow for self-reporting versus only including PCR-tested confirmed individuals, on the number of symptoms for which patients are queried to allow classification as Long COVID, and on the context, such as the vaccination rate. The UK Office for National Statistics reported a prevalence rate of approximately 3% (12 symptoms queried) [5]. Another very large study found a prevalence rate of approximately 22% (26 symptoms queried) [6]. A meta-analysis of 194 published studies combining confirmed and self-reported cases and allowing for 1 symptom queried suggested as many as 45% of COVID-19 survivors, regardless of hospitalization status, had a range of unresolved symptoms at 4 months [7]. Indeed, while self-reporting may risk over-diagnosis, the ONS findings may actually underestimate the true prevalence. 

Post-COVID-19 syndrome or PASC has been described by the World Health Organization as the persistence of symptoms or new symptoms more than 30 days post-SARS-CoV-2 infection. The diagnostic criteria for post-COVID-19 syndrome [8] are shown in the Box below (Box 1).

Box 1 **Diagnostic criteria for post-COVID-19 syndrome or PASC according to the WHO or Long COVID according to NICE.**

(1) Persistence of symptoms or new symptoms more than 30 days post-SARS-CoV-2 infection. Post-COVID-19 condition occurs in individuals with a history of probable or confirmed SARS CoV-2 infection, usually 3 months from the onset of COVID-19 with symptoms that last for at least 2 months and cannot be explained by an alternative diagnosis.
(2) Common symptoms include fatigue, shortness of breath and cognitive dysfunction, but also others, and generally have an impact on everyday functioning.
(3) Symptoms may be new onset following initial recovery from an acute COVID-19 episode or persist from the initial illness. Symptoms may also fluctuate or relapse over time.

Specified further as:

(4) Acute and persisting: Occurring 0–30 days post-COVID-19 PCR and persisting 30–120 days post-test.
(5) Late: Occurring initially 30–120 days post-test.

According to NICE, in addition to the clinical case definitions, the term ‘long COVID’ is commonly used to describe signs and symptoms that continue or develop after acute COVID-19. It includes both ongoing symptomatic COVID-19 (from 4 to 12 weeks) and post-COVID-19 syndrome (12 weeks or more) [1].

As the specific pathophysiology of Long COVID has not yet been clarified, there are no definite criteria of the condition, hence the World Health Organization’s definition is quite broad [9]. Moreover, other countries may use different criteria in terms of required symptoms and duration. The lack of application of common diagnostic criteria for Long COVID contributes to the existing controversy about the true prevalence of Long COVID. In addition, Long COVID is a markedly heterogeneous condition [10,11,12,13], which may hinder recognition. In a large analysis exploring phenotypes conducted over 10 cohorts, 31 clinical symptoms were identified. After fatigue and breathlessness-related symptoms, difficulty in walking ranks as the most reported symptom (>25%) [14]. Balance problems are reported in about 20% of Long COVID patients [15].

Whereas symptoms such as brain fog, anxiety and low mood are readily accepted as symptoms of Long Covid [16,17], reports are emerging in social media [18], and recognized by clinicians in personal communications, that patients with motor and balance problems after COVID-19 infection experience their symptoms being mislabeled as Functional Neurological Disorder (FND) in the absence of proper testing. 

FND, also known as conversion disorder in the DSM-5-TR [19], or as dissociative neurological symptom disorder in the ICD-11 [20], is characterized by the presentation of motor or sensory symptoms that lack compatibility of the symptom with a neurological substrate. There are several subtypes: motor function, sensory function, non-epileptic seizures, speech problems, problems with the senses and mixed type. The main DSM-5-TR criteria are shown in the box below (Box 2).

Box 2 **DSM-5 criteria for functional neurological disorder.**

1. One or more symptoms of altered voluntary motor or sensory function.
2. Clinical findings provide evidence of incompatibility between the symptom and recognized neurological or medical conditions.
3. The symptom or deficit is not better explained by another medical or mental disorder.
4. The symptom or deficit causes clinically significant distress or impairment in social, occupational or other important areas of functioning, or warrants medical evaluation.

FND has been the subject of academic and clinical debate for centuries, with limited understanding of the underlying mechanism, diagnostic methods and effectiveness of treatment [21]. Although the diagnosis is dependent predominantly on the exclusion of any other medical condition that could account for the symptoms according to the ICD-11 and DSM-5-TR, current neurological practice of FND classification allows for such comorbidity, for example in case of motor symptoms in Long COVID patients. In this context, the interpretation of how to deal with such concomitant symptomatology, and how to conceptualize, diagnose and treat them, poses a clinical challenge. We aim to explore aspects of this challenge from the clinician, patient and caregiver perspective.

## 2. Materials and Methods

In this article we will use the term Long COVID, as this is used by the patients in the vignettes and has been adopted by NICE, but in case of citations using the other terms we will use post-COVID-19 syndrome or PASC as well.

The STIMULATE-ICP study is the largest clinical study of Long COVID to date, funded by NIHR, that is currently ongoing. A major new consortium of researchers, health professionals and patients are engaged in it to conduct a clinical trial evaluating integrated care and medication for Long COVID. Still in its recruitment phase, currently more than 500 patients have been recruited and are participating in the trial [22]. In addition, the STIMULATE-ICP-Delphi sub study seeks to answer the research question what are effective integrated care pathways for individuals with Long COVID and how can they be transferred to other long-term conditions [23]. This NIHR-funded research programme will inform medium and longer-term policy and health system responses. The patient participant involvement (PPI) group involved in STIMULATE-ICP [24] encompasses 30 people with lived experience of Long COVID, 13 of whom contributed to the study in a series of eight engagement events and 3 of whom reported their personal experiences. 

The CANDO study is a program of research aiming to understand more about the causes of FND so that new therapeutics may be explored in the future. An observational feasibility study in 15 patients explored whether people with FND might have high levels of inflammation in their blood, reported a lot of stressful experiences, showed signs of long-term stress, and showed any cognitive symptoms such as problems with attention, concentration or memory [25]. A feasibility pilot indicated elevated biomarkers for systemic low-grade inflammation [26], and currently an experimental study is being prepared to explore treatment modes for this. The patient participant involvement (PPI) group involved in CANDO encompasses 31 people with lived experience of FND, 18 of whom described issues related to service delivery and diagnosis of FND; 2 of these 18 patients contributed to this study by reporting their experiences.

Excerpts with direct quotes from the reports provided by PPI from both groups were used to illustrate this clinical challenge, as demonstrated by vignettes of experiences of patients and caregivers below. Terms as Long COVID and FND were used by patients themselves. The patients provided consent for the inclusion of the vignettes about their experiences. 

Clinician input was taken from personal communications and the literature, and is provided in the text.

## 3. Results

### 3.1. Diagnostic Classification

Clinicians indicate that an FND diagnosis in Long COVID without proper testing for Long COVID-related mechanisms may be a misdiagnosis, and that such symptoms may actually be attributable to pathology linked to Long COVID. They express concerns regarding how many patients have been seen and quickly labelled with FND, a label that later turns out to be incorrect. They emphasize how ‘good neurologists or other physicians of any kind would not diagnose functional problems without good evidence such as positive physical signs’. This refers to current efforts to establish positive indicators of the diagnosis of FND, by recognising certain signs during neurological examination. Examples include the Hoover sign, described for the first time by Charles Franklin Hoover in 1907 as involuntary extension of the ‘normal’ leg that occurs when flexing the contralateral leg against resistance. This relies on the principle of synergistic contraction. It shows that muscle groups can still work, however not during an intentional movement of the patient, but as part of an unintentional antagonistic movement accompanying an intended contralateral movement, for example in the hip [27]. Sensitivity of the Hoover test is limited to 63%, but specificity is 100% [28]. Gradually, the use of this sign to establish a diagnosis of FND is becoming more widespread [29]. However, this requirement for a positive finding during neurological examination is not a criterion for FND in DSM-5-TR or in ICD-11. Moreover, this approach may not be applicable in all types of FND, such as with vertigo or dizziness, or psychogenic non-epileptic seizures (PNES). Given that postural orthostatic tachycardia syndrome (PoTS) is so common in Long COVID [30,31], diagnosing FND in such cases should best be avoided or treated with caution.

### 3.2. How Diagnostic Processes Take Place

Currently, there remains no clinically utilized confirmatory test for Long COVID. High-resolution MRI scans and PET scans, amongst others, are being explored, but do not yet feature in the clinical evaluation of patients presenting with symptoms of Long COVID, and this contributes to no explanations being found in Long COVID patients presenting with motor symptoms. Given the broad WHO criteria and the lack of conclusive diagnostic tests, it is difficult to correctly diagnose Long COVID, and there are currently no clear diagnostic methodologies or treatments for Long COVID. In addition, the diagnostic criteria for neurological complications of PASC, including ‘brain fog’, ‘memory deficits’ and ‘encephalopathy’ are not established, which makes PASC-associated neurological syndromes impossible to consistently classify [9,32]. 

Consequently, given that neither FND nor Long COVID has a definitive test, an FND diagnosis can lead to no access to Long COVID-related care and patients being dismissed despite some similarities in the presentation and ongoing symptoms. Other factors at play can be that dysautonomia, such as PoTS [33], which are very common in Long COVID [34], require expert knowledge to differentiate with FND. 

A patient says:

‘I had COVID in April 2020 and have Long Covid experiences since. I was not referred to a Long COVID clinic, they do not exist where I live. I was referred to Neurology in December 2020, following onset of tremors, balance and reduced sensation; they did basic reflex and walking tests only and diagnosed me with FND. When I saw cardiology for tachycardia and PoTS symptoms in 2021, they did cardiac function, but no recognised PoTS tests, like tilt table etc. I was presented at the emergency department after a seizure-type event in March 2022, and they did not refer me to the first fit clinic, which is against national guidance, but only wrote a note to Neurology, stating, without having performed a scan or EEG, that I had FND. I finally saw neuropsychiatry in October 2022, where, again, they only did reflex and walking tests and insist I have FND. I did argue my way to MRI scans, but do not have the results. Despite SIGN guidance, they are not ordering EEG. I feel like my Trust has a policy of diagnosing FND in Long Covid patients, without doing the proper diagnostics to exclude other things, especially dysautonomia conditions’.

It may seem surprising in itself that A&E doctors or other non-specialists would make such a complex diagnosis. Misdiagnosis of a medical condition as FND was not a rare phenomenon even before the start of the COVID-19 pandemic [35]. However, neurologists have also been reported to diagnose FND in Long COVID patients. This point warrants further exploration to understand the extent of primary care and non-specialist diagnoses of FND, and revisiting the idea of comorbidity in FND. The need for this is more pressing now that FND diagnosis can risk people with Long COVID missing out on specialist input as a result of inappropriate diagnosis. 

A caregiver says:

‘My 40-year-old wife had Covid which resulted in breathing difficulties, becoming unresponsive and being blue-lighted to hospital. Whilst in hospital she started having near-constant and high-magnitude involuntary movements… She was discharged within 24 h and we were told the shaking should go in a few days, but this hasn’t been the case. This has caused her to have significant difficulties with walking… Over several months, she saw a private neurologist after the NHS one initially declined to see her. They don’t think it’s an organic neurological issue but think it may be FND. It may well be FND but given the rapid onset and the link to it starting being her having COVID it seems that there must be some link there which our current neurologist isn’t exploring and the GP is disinterested now there is a FND diagnosis’.

Patients say:

‘I have Long COVID. The neurology clinic attempted to characterize my symptoms of dizziness and exertional tachycardia as functional following a four-minute-long telephone appointment. Tilt-table testing elsewhere subsequently showed I have initial orthostatic hypotension…This misdiagnosis affected my ongoing access to medical care and ability to get referrals’.

‘I asked for a second opinion who overturned the diagnosis and referred me to a Long COVID clinic and a Fatigue Clinic. However, both refused me based on the erroneous FND diagnosis’.

This also happens in children. A parent caregiver says:

‘Our 12 year old daughter caught COVID in July 2021 and was left with parosmia. Around Christmas she developed dizziness, chest pains and daily fainting episodes. She has seen a neurologist and had multiple tests, which [were all reported as normal]. The neurologist said it is psychogenic non epileptic seizures (PNES) and referred her to a psychiatrist, who said on the phone that she doesn’t think she can help as she hasn’t come across anyone with this before. Our daughter was a happy, sporty otherwise healthy child. She’s never had any trauma, abuse, bullying, bereavement, PTSD to deal with in life. We tried to get a Long COVID clinic referral but were advised a referral to CAMHS. We were disappointed as we know she has Long COVID as she still has parosmia which they didn’t mention’.

### 3.3. Pathogenetic Pathways

The pathogenesis of Long COVID is still unclear and probably multifactorial [36]. Possible pathways are neuroinflammation by increased cytokines, inducing reactive states of microglia; invasion of the central nervous system entering neurons and glial cells [37] with recently virus RNA having been found in a brain autopsy of one post-COVID-19 patient as late as 230 days following symptom onset [38]. It may be an autoimmune response [39]; or reactivation of latent viruses such as Herpes and Epstein Bar Virus, triggering neuropathology [40]. Walking and balance may be affected by imaging-observed reductions in grey matter thickness and reduction in global brain size after infection with SARS-CoV-2; and additionally by changes in limbic regions, emotional state, and cognitive decline [41]. Other possible explanations include general fatigue, which appears to be associated with chronic neuroinflammation [37]; medication side effects, metabolic disturbances, cardiac involvement, as well as myopathy, muscle pain and arthralgia [14], dysautonomia, respiratory dysregulation [42], and coagulopathies such as micro-clot formation [43].

There are reports suggesting lingering virus as a potential cause of Long COVID [44,45]. For most infected people, virus levels in the body peak between three and six days after the original infection [46], and the immune system clears the pathogen within 10 days. However, persistent viral shedding 14 days after initial testing was found in 42% of hospitalized COVID-19 patients and was associated with increased 6-month mortality [47]. And after more than 90 days, 12 percent of the persistent shedders were still testing positive; one person tested positive 269 days after the original infection. A case study reported persistent viral shedding in a man with mild persistent symptoms after 60 days [48]. Systemic viral persistence has been detected via reverse transcription-polymerase chain reaction (RT-PCR) and within subgenomic RNA has been demonstrated beyond 10 days in non-immunocompromised individuals [49] and is considered a mechanism resulting in a prolonged and debilitating symptom profile [50]. Furthermore, in a sample of 317 patients diagnosed with COVID-19, high serum levels of IL-17 and IL-2 and low levels of IL-4 and IL-10 appeared to constitute a cytokine profile of long COVID-19 [51].

Properly diagnosed FND, paying attention to rule-in symptoms, has a growing evidence base of a biological underlying mechanism. Indeed, there is evidence for structural as well as functional changes in FND in brain MRIs and the discussion how this relates to FND, to other neurological conditions and to psychiatric comorbidity is ongoing [52]. Elevated cytokines such as IL6, IL17 and TNFa indicative of Systemic Low-grade Inflammation (SLI) have been found in FND patients [53].

### 3.4. The Interface of Long COVID, Mental Disorders, and FND

At the interface of ongoing somatic and psychiatric symptoms and mechanisms, people with neurodevelopmental disorders such as autism spectrum disorder, ADHD and hypermobility seem to be more susceptible to develop Long COVID. It is uncertain whether these risk factors represent shared biological susceptibility, vulnerability or diagnostic overlap. On top of biological mechanisms as described above, anxiety and depression may be provoked by the debilitating symptoms and increase the symptomatology. Furthermore, lack of knowledge among patients, clinicians and the general public regarding how to achieve recovery in Long COVID may be sustaining factors in Long COVID motor and balance problems.

There have been suggestions that Long COVID may be purely functional (i.e., psychological) in origin [54] and it is possible that ’diagnosing’ patients with Long COVID with FND is symptomatic of that line of thought. However, although FND, if properly diagnosed, is likely a valid clinical disorder, it is often over-diagnosed without paying attention to rule-in symptoms, especially in vulnerable and underserved populations [21]. And although a study found that people with pre-infection anxiety or depression had increased risk of self-reported post-COVID-19 conditions [55], this is a phenomenon that can occur in medical conditions more generally, also conditions that are not considered to be functional in nature. For example, experiencing a higher burden of disease occurs in patients with comorbid depression in Diabetes Mellitus, who tend to perceive a higher diabetes symptom burden [56]. There have however been so far no data corroborating that Long COVID is a functional disorder in the sense of caused by psychological nonorganic mechanisms alone. Furthermore, little is known about patterns of recovery in Long COVID and it is striking how patients do not get access to proper rehabilitation programs despite severely impaired functioning.

### 3.5. Impact on Functioning

General functioning is severely impaired in patients with Long COVID. Impaired exercise capacity such as in post-exertional malaise (PEM) has been reported to occur in 20% of patients, of which 18–55% was found to have indicators of a hypercoagulable state in lab tests [57]. Physical activity may worsen the condition of 75% of Long COVID patients and improves less than 1% [22], which suggests possible ongoing microvascular or endothelial dysfunction to play a role. Therefore, antithrombotic therapy might be considered in the treatment of these patients, which is currently evaluated in the ongoing STIMULATE-ICP trial [58]. So far, however, this is not clinical practice. Regarding the impact of their symptoms on functioning, patients say:

‘I was diagnosed with FND for Long Covid symptoms. At this time I could not walk further than about 50 meters and I could only take the stairs once a day. The neurologist explained that my symptoms were all due to anxiety and referred me to talking therapy’.

‘After the FND diagnosis I was left without access to any support other than my GP despite my ongoing problems that to me seem Long COVID related. My GP is desperate as she wants to refer me for treatment but does not know how as having the FND diagnosis makes me ineligible for referrals to treat Long COVID’.

‘The treatment provided by the physiotherapist for the erroneous FND diagnosis included an insistence on physical activity and exercise regardless of my tolerance levels. My condition deteriorated significantly and I am now mostly bedbound’.

A caregiver says:

‘My wife’s current condition is having a huge impact on our lives and those of our 2 young children. Before having COVID she was fit and healthy and had no underlying health issues. She was a high-flying professional, the most sociable person you’re ever likely to meet and talked at a rate of knots and now she can’t really walk, struggles to talk and constantly twitches and jerks. This all makes her feel self-conscious and withdrawn’.

### 3.6. Prognosis

As a disease, Long COVID is not an altogether innocent condition. For example, a recent study in the USA found that between 1 January 2020 and 30 June 2022, 3544 people with Long COVID died, which is 0.3% of the 1,012,487 deaths with COVID-19. In 67.5% of cases, COVID-19 was mentioned as an underlying or contributing cause of death on the death certificate [59,60]. This warrants providing treatment for patients presenting with clinical symptoms such as walking, balance and PNES problems after COVID-19 infection.

Similarly, mortality is increased in FND, especially in cases of PNES. A 2020 study found that mortality in PNES was 8.2%, 2.5 times higher than the standardized mortality ratio of the general population and roughly similar to the rate in patients with epilepsy (9.4%) [61]. This finding was confirmed in a recent study, which found a hazard ratio for natural deaths of 8.1 for PNES compared with the general population [62]. A professor of neurology commented that these data would ‘hopefully help neurologists to take this disorder more seriously, learn to diagnose patients as early as possible, and follow patients until they engage in treatments’ [63]. This underscores the importance of treatment being provided.

### 3.7. Addressing Clinical Need

In view of the diagnostic ambiguity, there seems to be an urgent clinical need to deal with the differential diagnostic and treatment issues that arise in patients with Long COVID experiencing motor and balance problems. What would be needed for that? Access to proper diagnosis and treatment by clinicians with knowledge of both conditions seems to be key.

A patient says:

‘I’m not sure really, as even now I’m not getting appropriate support from the NHS. Everyone is so firmly of the view that it’s FND that nothing else is being looked at and all I get told is that it’s FND and it’ll only get better if I accept the diagnosis and have CBT, physio and speech therapy. I’ve had all those treatments and they’ve not improved things at all... I’ve been signed off by all as there is nothing they can do. There is no underlying trauma/stress/anxiety to address and all professionals I’ve seen agree with this, and so I’m not a ‘classic’ FND patient and the usual treatments are not working but there is no looking at what else it could be’. 

‘The support I have needed from the NHS is a bit of curiosity and thinking outside the box to consider the actual underlying causes and how they might be addressed. Instead all I get told is that as it’s FND, there’s little to be done and there is no need to look into it further’. 

A parent caregiver says:

‘I feel like many medical professionals aren’t aware of PNES / NEADS / FND. They certainly aren’t aware that there is a link between children developing FND post covid. I feel like I have had to fight for nearly a year to get someone to agree that COVID triggered all of our daughter’s symptoms including non-epileptic seizures… There are so many children on the Long COVID Facebook groups who have been diagnosed with FND and we were all told that it was due to anxiety (from lockdown, from being a teenager etc).... You have to fight for tests to rule out other issues... There can’t be all these children suddenly with seizures, paralysis, muscle weakness after COVID just by coincidence’.

## 4. Discussion

### 4.1. Clinical Implications

The NHS has commissioned 90 specialized Long COVID clinics and supports major research efforts to develop treatments [64,65]. However, in the absence of accurate diagnostic tests or proven treatments for both Long COVID and FND, greater focus must be given to exclude alternative diagnoses, and importantly ensuring patients feel supported, until a greater knowledge of both FND and Long COVID is established. Diagnostic approaches should take biological underpinnings as well as possible psychological mechanisms and the patient perspective into account [23]. Education of clinicians in the heterogeneous manifestation of Long COVID and diagnosis of FND may be needed. Patients could potentially benefit from treatment of their motor symptoms in Long COVID clinics, to follow rehabilitation and other programs, combined with psychiatric treatment as needed. Given the experiences of patients, their caregivers and clinicians, instead of dismissing patients with a possibly misdiagnosed FND label for potentially Long COVID-related motor symptoms, integration of diagnosis and treatment should be given priority.

### 4.2. Research Implications

Research into underlying mechanisms for motor and balance symptoms in Long COVID should be undertaken. This should also explore the question of how to determine whether motor and balance symptoms currently diagnosed as FND should be considered one part of Long COVID symptoms, in other words, one component of symptomatology. Such research should take into account how the diagnosis of such symptoms as part of Long COVID could be made, and how they differ from validated ways to establish the diagnosis FND.

By definition, according to the DSM-5TR and the ICD-11, no alternative condition should be able to explain FND; importantly this would include a diagnosis of Long COVID. Moreover, the WHO classification of post-COVID-19 syndrome does not allow for explanation of the symptoms by another medical condition. Given the current practice, research should explore how an FND classification is a possible confounder of diagnosing Long COVID, and how the diagnostic criteria for neurological complications in Long COVID, including ‘brain fog’, ‘memory deficits’ and ‘encephalopathy’ could be improved to include and properly classify motor and balance symptoms. This would require a rethink of the tendency to diagnose FND as a comorbid condition in other medical conditions that might explain the symptoms. That goes against classification criteria and should be avoided.

Research should also explore how often FND has been diagnosed in Long COVID, and what could be the pathways driving FND, Long COVID and treatments of these two as distinct disorders.

Research should explore possible underlying mechanisms and biomarkers in both Long COVID and FND where there is similar symptomatology. This could inform further research into possible treatments such as cytokine blockers or antiviral medication in Long COVID, and cytokine blockers in FND. In terms of treatment, the STIMULATE-ICP-Delphi study explores what integrated care for Long COVID and other LTCs should look like [23,66], and this can include how the treatment of Long COVID with motor and balance problems might be designed.

Research should address the lack of a shared cognitive paradigm among patients and clinicians regarding rehabilitation from a serious infection including Long COVID. We live in an era of availability of antibiotics that have changed medical practice greatly in how recovery is addressed. Most serious infections can be treated with them, and people therefore recover much more swiftly than before. For example, before the availability of antibiotics, people would suffer from tuberculosis and the socially affluent would go to sanatoria for months or years to give their body rest, and relying on sunshine, good food, friendly conversations, exchanging pleasantries and light exercise to recover. They would not work or perform household chores for a long time. Clinicians and nurses in such clinics would know very well how to balance those activities and rest in order to support gradual recovery. However, knowledge of that practice has slipped away and needs to be reinvented and placed in current health and social care settings by research. Moreover, it has to be made available for all social classes, so such research should take social inequalities into account. Furthermore, research should take the psychological aspects of recovery into account. Such research might also be relevant for chronic conditions of which the origin is not well known, such as FND. Such a programme of research would enable policymakers to consider options for policies.

## 5. Conclusions

Labeling neurological dysfunction such as walking, balance and other neurological dysfunctional problems in Long COVID as FND without proper testing of both conditions does not serve patients who are then erroneously diagnosed with FND and dismissed from Long COVID services. Research into underlying mechanisms and diagnostic methods should explore how to determine whether motor and balance symptoms currently diagnosed as FND should be considered one part of Long COVID symptoms, in other words, one component of symptomatology, and in which cases they actually correctly represent FND. Research into rehabilitation models, treatment and integrated care is needed, and should take into account biological underpinnings as well as possible psychological mechanisms and the patient perspective. 

## Data Availability

No new data were created or analyzed in this study. Data sharing is not applicable to this article.
